# MedSpectralNet: A lightweight convolutional neural network architecture for multi-modal image classification

**DOI:** 10.1371/journal.pone.0346128

**Published:** 2026-04-27

**Authors:** Nabilah Afrin, Masud An-Nur Islam Fahim, Wasan Alamro, Yazan M. Allawi, Ahmad Abadleh, Salman Md Sultan, Ersin Elbasi, Aymen I. Zreikat

**Affiliations:** 1 Innovative Skills Ltd., Dhaka, Bangladesh; 2 Department of Communications and Computer Engineering, Faculty of Engineering, Al-Ahliyya Amman University, Amman, Jordan; 3 Department of Electrical Engineering, College of Engineering, Princess Nourah bint Abdulrahman University, P.O. Box 84428, Riyadh 11671, Saudi Arabia; 4 CS Department, Mutah University, Karak, Jordan; 5 College of Engineering and Technology, American University of the Middle East, Egaila, Kuwait; University of Manitoba, CANADA

## Abstract

Medical image classification requires models that effectively capture both fine-grained local patterns and global anatomical structures while maintaining computational efficiency for clinical deployment. Although state-of-the-art models such as MedMamba utilize State-Space Models (SSMs) to balance accuracy and efficiency, their sequential operations limit parallelism and increase runtime. To overcome these limitations, we propose MedSpectralNet, a lightweight Convolutional Neural Network (CNN) architecture that approximates self-attention with linear complexity to efficiently extract multi-frequency features. The model introduces a dual-stream feature extractor that processes global and local information in parallel, and a ContextGate block that adaptively fuses multi-scale representations. MedSpectralNet is evaluated across six benchmark datasets from MedMNIST (including BloodMNIST, BreastMNIST, DermaMNIST, PneumoniaMNIST, OrganCMNIST, and OrganSMNIST), MedSpectralNet achieves an average accuracy of 93.7% on OrganCMNIST and 98.0% on BloodMNIST, showing 1–4.3% relative accuracy gains when compared to larger transformer-based models. Importantly, it delivers this performance with only 8.5 million parameters, representing approximately 60% fewer parameters than MedMamba-T, which requires 14.5 million parameters. MedSpectralNet has also achieved high AUC values up to 0.999 across multiple classes, demonstrating state-of-the-art accuracy with substantially reduced computational cost and improved parallelization, which makes MedSpectralNet well-suited for real-time and resource-constrained classification-based medical applications.

## 1 Introduction

Medical image classification represents a critical component of modern healthcare diagnostics. It requires computational models that are capable of extracting features from multiple levels of the images. The efficiency of the models relies on capturing low-level features, such as textures, edges, and pixel intensities that reveal anatomical boundaries, as well as high-level semantic features like organ shapes, tissue types, and pathological signs needed for accurate diagnosis [1–4].

Apart from accurate performance, the diagnosis system demands attention to computational efficiency for practical deployment. This efficiency requirement becomes particularly critical given that healthcare settings often operate with limited computational resources, requiring models that deliver strong performance while maintaining low memory usage and fast inference speeds [5,6]. Furthermore, real-time diagnostic tools must prioritize efficient processing to ensure they integrate seamlessly into clinical workflows without causing delays that could impact patient care.

Deep learning has demonstrated remarkable success in medical image analysis across diverse applications. Convolutional Neural Networks (CNNs) have proven particularly effective in lung imaging tasks, including nodule detection in chest X-rays and CT scans, where their ability to capture fine-grained textural patterns enables accurate identification of pulmonary abnormalities [1,7–10]. Beyond pulmonary imaging, CNN-based approaches have achieved strong performance in breast cancer classification from ultrasound images [11] and bladder lesion segmentation in MRI [12]. Vision Transformers (ViTs) have further advanced the field by modeling long-range spatial dependencies, showing promise in whole-slide pathology image analysis and multi-organ segmentation tasks [13]. These architectural innovations have established CNNs and ViTs as state-of-the-art approaches on standard medical imaging benchmarks such as MedMNIST [13], demonstrating the transformative potential of deep learning in clinical diagnostics.

CNNs excel at extracting local features, such as fine-grained tissue textures or lesion boundaries, which are crucial for detecting subtle pathological indicators [14]. However, their limited receptive fields hinder their ability to capture long-range dependencies, such as spatial relationships between anatomical structures, which are essential for holistic diagnostic accuracy in complex medical images [15]. In contrast, ViTs use self-attention mechanisms to model global context effectively. But their quadratic computational complexity, *O*(*NC*(*HW*)), for image dimensions H×W, renders them computationally prohibitive for high-resolution medical scans, particularly in real-time or resource-constrained clinical settings [2]. As medical imaging data grows in resolution and complexity, models must balance contextual understanding with computational efficiency for practical clinical deployment [16]. Recent lightweight CNN architectures have also made notable progress in balancing efficiency and performance for medical image classification. MobileNetV4 [17] uses universal inverted bottleneck (UIB) blocks combined with Neural Architecture Search for efficient feature extraction. FastViT [18] introduces RepMixer tokens for efficient spatial mixing, EdgeNeXt [19] employs split depth-wise transpose attention to improve efficiency, and EfficientFormerV2 [20] shows that well-designed attention mechanisms can achieve both speed and accuracy.

Despite these innovations, current architectures face specific limitations in medical imaging. First, they typically use standard downsampling that fails to account for the multi-frequency nature of medical data, where both fine textures and coarse structures are diagnostically relevant. Second, existing depthwise separable convolutions lack adaptive mechanisms to prioritize clinical patterns over background noise.

To mitigates, researchers have proposed hybrid architectures and State-Space Models (SSM). The Mamba architecture [5], for example, provides a linear computational complexity of O(HW) while successfully capturing long-range dependencies. However, the sequential nature of SSMs makes them harder to parallelize at a large scale, which can increase runtime and reduce utilization of modern massively parallel GPUs. Thus, while SSM-based models achieve an efficiency-accuracy trade-off, they face challenges in throughput and latency [6,16].

To address these trade-offs, we propose MedSpectralNet, a lightweight convolutional architecture designed for medical image classification. The architecture focuses on efficient hierarchical feature extraction while leveraging parallel processing capabilities of modern hardware architectures. The main contributions of this paper are as follows:

- Introduce a CNN-based SpectralFlow with ContextGate, a lightweight convolutional module that achieves self-attention-like performance with linear complexity *O*(*HW*). The module divides features into high-frequency and low-frequency streams to capture detailed information. It uses ContextGate for adaptive fusion of multi-scale patterns while maintaining minimal computational and memory requirements.
- Present a dual-stream feature extraction framework that processes high-frequency local details and low-frequency global context through parallel pathways. This design preserves complementary information without creating sequential processing bottlenecks. Thus, it enhances robustness across variations in texture, shape, and intensity.
- Conduct comprehensive empirical validations across six medical imaging datasets from the MedMNIST benchmark to demonstrate computational efficiency. Our findings show that MedSpectralNet achieves comparable performance to MedMamba-T while utilizing significantly fewer parameters and requiring reduced computational resources. This enables faster and more parallelizable inference suitable for practical deployments.

The remainder of this paper is organized as follows. Section 2 reviews related work on medical image classification. Section 3 details the MedSpectralNet architecture, including the SpectralFlow Module and ContextGate Block. Section 4 presents experimental results for applying the proposed MedSpectralNet on the MedMNIST benchmark datasets, discussing the implications for its clinical deployment, and Section 5 concludes the study with future research directions.

## 2 Related works

The field of medical image classification has witnessed rapid advances in recent years, driven largely by the adoption of deep learning architectures. The effectiveness of deep learning extends across multiple medical imaging domains and anatomical regions [1,9,10,12]. In pulmonary imaging, CNNs have demonstrated strong performance for pneumonia detection from chest X-rays, with recent work showing that compact models can achieve clinically meaningful results even in resource-constrained settings. In abdominal imaging, automated kidney lesion classification has similarly benefited from lightweight architectures that balance diagnostic accuracy with computational efficiency. These domain-specific applications highlight the need for tailored architectures that accommodate the unique characteristics of different imaging modalities while remaining practical for deployment.

Early approaches relied primarily on CNNs, which proved highly effective at capturing local features such as tissue textures, lesion boundaries, and subtle anatomical variations, yielding strong results across diverse clinical datasets. For example, one study combined a multi-stage preprocessing pipeline with a ResNet50 backbone for brain tumor detection using the Figshare MRI dataset, demonstrating how preprocessing techniques such as Gaussian smoothing, bilateral filtering, and K-means segmentation can enhance CNN-based diagnostic accuracy [14]. Despite such development, CNNs are inherently limited by their restricted receptive fields, making it difficult to model long-range dependencies that are critical for holistic interpretation of complex medical scans.

To overcome these limitations, ViTs have been introduced as an alternative, offering the ability to capture global contextual information across entire images. A representative framework, Med-Former [21], employed parallel attention paths with varying window sizes to extract multi-scale features, while also incorporating a Spatial Attention Fusion (SAF) module to mitigate information loss through selective feature fusion. These designs highlight the ability of transformers to model complex relationships across anatomical structures. However, the quadratic computational complexity of self-attention remains a significant drawback, particularly for high-resolution medical images where real-time or resource-constrained deployment is required.

Recent efforts have therefore shifted toward hybrid frameworks and SSMs, which combine the strengths of CNNs, transformers, and sequential architectures to balance efficiency and accuracy. One such approach, Vision Mamba [5], integrates convolutional layers with structured SSMs to capture both local details and long-range dependencies. The architecture utilizes grouped convolutions and channel shuffle operations to preserve linear computational complexity while maintaining competitive performance across multiple imaging modalities. This demonstrates the potential of SSM-based methods to achieve transformer-like accuracy at a fraction of the computational cost.

Beyond specific classification architectures, recent literature demonstrates the versatility of hybrid and attention-based deep learning across various medical domains. For instance, in segmentation tasks, bilateral collaborative streams with multi-modal attention networks have shown significant promise for accurate polyp segmentation [22], highlighting the importance of feature fusion. Similarly, the integration of CNNs with attention mechanisms has been effectively applied to magnetic resonance imaging (MRI) for improved brain tumor classification [23], corroborating the utility of attention in capturing subtle anatomical variances.

Hybrid CNN-Transformer designs have also gained traction for medical image classification. For instance, some models replace standard self-attention with more efficient convolutional attention mechanisms [24], thereby reducing quadratic complexity while simultaneously improving adversarial robustness through augmented feature regularization. Evaluations on the MedMNIST benchmark [13] have shown that such architectures generalize well and achieve strong robustness under lower overhead. Other frameworks extend this line of work by integrating multi-core and depthwise-separable convolutions with selective scanning modules, enabling linear complexity modeling of both local and global context [25]. More recent advancements have embedded functional layers such as Kolmogorov–Arnold Networks (KAN) [26] and adopted dilated neighborhood attention mechanisms to expand contextual perception, striking a refined balance between efficiency and generalization.

In the realm of biomedical signal processing, robust feature learning approaches have been developed for epileptic seizure detection [27] and prediction using multi-feature fusion in IoT frameworks [28]. Furthermore, novel expert systems for heart disease diagnosis [29,30] and AI-driven hybrid neural networks like CardioGuard for ECG authentication [30] demonstrate the growing trend of deploying efficient, high-performance models in telehealth and clinical decision support systems. These works collectively underscore the shift towards architectures that high-level semantic understanding with computational robustness.

Beyond architectural innovations, there is also a growing emphasis on lightweight solutions tailored for specific clinical tasks. Studies in areas such as pneumonia detection and kidney lesion classification have demonstrated that compact models can achieve clinically meaningful performance while remaining suitable for deployment in settings with limited computational resources [7,8]. These findings underscore the importance of designing architectures that deliver accuracy, efficiency, scalability, and ease of integration into real-world healthcare systems. Building on these insights, this paper introduces MedSpectralNet, a lightweight convolutional architecture that incorporates spectral feature decomposition and adaptive contextual modeling to capture both local and global features efficiently, ensuring competitive diagnostic accuracy while substantially reducing computational cost, thereby addressing the practical constraints of clinical deployment.

## 3 Methodology

The proposed MedSpectralNet architecture is developed as a lightweight yet powerful framework for medical image classification. Its design directly addresses the computational demands of high-resolution imaging, where models must extract rich diagnostic cues without overwhelming available resources. To achieve this, MedSpectralNet emphasizes a careful balance that preserves the ability to capture fine local details such as tissue boundaries or micro-lesions, while also modeling broader anatomical context that is critical for accurate diagnosis, all within a computationally efficient structure.

At the core of the architecture are three complementary elements. The SpectralFlow module, which serves as a mechanism for adaptive spatial feature processing, decomposes inputs into high- and low-frequency components to highlight both subtle textures and large-scale patterns. The ContextGate block, which introduces dynamic modulation, selectively enhances the clinically relevant features while suppresses noise and redundant information, much like a gatekeeper prioritizing the most useful signals. Finally, a residual backbone that ensures stable information flow and preserves key representations across layers, preventing the loss of crucial diagnostic details. Together, these components form a streamlined and expressive model, tailored to handle complex medical images and capable of supporting efficient clinical deployment.

Table 1 summarizes the key mathematical symbols, feature representations, intermediate tensors, and evaluation metrics used throughout the proposed framework.

**Table 1:** Summary of symbols, meanings, and tensor shapes used in the proposed method.

Symbol	Meaning	Shape / Type
*x*	Input image	${\mathbb{R}}^{3\times H\times W}$
*H*, *W*	Image height and width	Scalars
*B*	Batch size	Scalar
*C*	Channel dimension	Scalar
*f*_*b*_	Backbone output feature map	${\mathbb{R}}^{B\times C\times H^{\prime }\times W^{\prime }}$ (*C*=512)
*f*_*c*_	Global pooled feature vector	${\mathbb{R}}^{C}$ or ${\mathbb{R}}^{B\times C}$
*k*	Patch size	Scalar (e.g., 3 or 5)
*x*_*l*_	Low-frequency after 1×1 conv	${\mathbb{R}}^{B\times (C/2)\times H\times W}$
*x*_*la*_	Attention-weighted low-frequency map	Same as *x*_*l*_
*x*_*h*_	High-frequency residual stream	Same as input stream
*a*_*l*_, *a*_*h*_	Learned per-stream attention weights	Broadcastable to stream shape
*x*_*f*_	Fused output of SpectralFlow	${\mathbb{R}}^{B\times C\times H\times W}$
*c*_*out*_	Spatial pathway output in ContextGate	${\mathbb{R}}^{B\times (C/3)\times H\times W}$
*g*	Gating tensor	${\mathbb{R}}^{B\times (C/3)\times H\times W}$
*i*	Identity pathway projection	${\mathbb{R}}^{B\times (C/3)\times H\times W}$
Concat(·)	Concatenation along channel dimension	Operator
⊙	Element-wise (Hadamard) product	Operator
*x*_*mod*_	Gated intermediate feature in ContextGate	${\mathbb{R}}^{B\times (2C/3)\times H\times W}$
*x*_*out*_	ContextGate projected output	${\mathbb{R}}^{B\times C\times H\times W}$
*x*_*final*_	Residual-added ContextGate output	${\mathbb{R}}^{B\times C\times H\times W}$

### 3.1 Overall architecture

**Fig 1:**
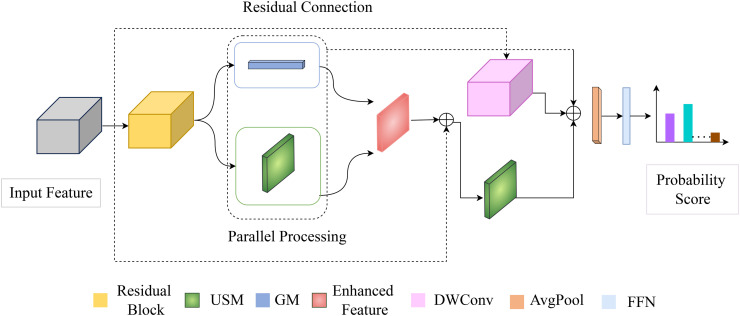
Schematic of the MedSpectralNet architecture with parallelizable convolutional operations.

The MedSpectralNet architecture, illustrated in Fig 1, processes an input medical image $x\in {\mathbb{R}}^{3\times H\times W}$ through a carefully orchestrated pipeline that prioritizes computational efficiency and diagnostic accuracy. The workflow begins with a ResNet-18 backbone [31] represented in the residual block, pre-trained on ImageNet [32] that extracts a base feature map $f_{b}\in {\mathbb{R}}^{C\times H^{\prime }\times W^{\prime }}$, where *C* = 512, *H*′ = *H*/32, and *W*′ = *W*/32. These features are preserved for residual connections to prevent information loss during subsequent processing. From this backbone output, MedSpectralNet branches into two parallel pathways. The first pathway, which we call the SpectralFlow pathway, is applied to decompose *f*_*b*_ into complementary high- and low-frequency components [33]. This pathway is denoted as an unidirectional spatial module (USM) in Fig 1. This decomposition enables the network to capture fine-grained local details such as tissue boundaries and micro-lesions, while simultaneously modeling coarse anatomical relationships like organ shapes and inter-structure context. This pathway ensures that both local and global features critical for medical diagnosis are effectively extracted. Concurrently, the ContextGate pathway, denoted as Gated Module (GM) in Fig 1, dynamically modulates feature channels and spatial locations, using a learned gating mechanism [34], to emphasize relevant features while suppressing noise or irrelevant information.

After parallel processing, the outputs of the two pathways are fused with the residual feature *f*_*b*_ using element-wise addition; this fusion preserves the backbone’s original information while enriching it with multi-frequency and contextually gated signals. Both pathways operate in parallel on the same input feature map *f*_*b*_, ensuring temporal synchronization without introducing sequential dependencies. Within this dynamic convolution operation, the low-frequency stream aggregates information over non-overlapping k×k patches through global average pooling and projection, while the high-frequency stream captures pixel-level details. By employing a k×k patch operation instead of a standard 3×3 convolution, the SpectralFlow Pathway leverages the difference in receptive fields to capture complementary information, extracting patch-level patterns dynamically.

A second SpectralFlow module further refines the fused map to improve spatial coherence, followed by batch normalization to stabilize training. The refined feature map is then aggregated via global average pooling into a compact vector $f_{c}\in {\mathbb{R}}^{C}$, and a final fully connected layer with softmax activation produces the classification scores. The entire architecture is designed for computational efficiency and achieves overall complexity on the order of $O(N\times C\times k^{2}\times H\times W)$.

### 3.2 SpectralFlow module

**Fig 2:**
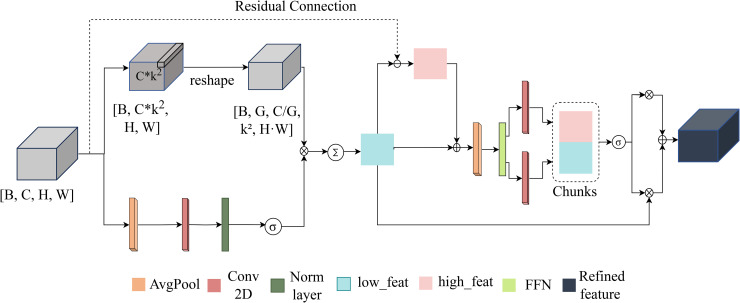
Structure of the SpectralFlow Module.

The SpectralFlow Module (Fig 2) is a lightweight convolutional mechanism designed to approximate the contextual modeling capabilities of self-attention while maintaining linear computational complexity.

Given an input feature map $x\in {\mathbb{R}}^{B\times C\times H\times W}$ from the backbone, the module first extracts global contextual information via adaptive average pooling, which aggregates features across the entire H×W spatial extent into a compact global receptive field $g\in {\mathbb{R}}^{B\times C\times 1\times 1}$. This is then transformed through a 1×1 convolution to generate position-specific filter coefficients that encode global contextual information.

Next, a 1×1 convolution reduces the channel dimension of *x*, producing a low-frequency representation $x_{l}\in {\mathbb{R}}^{B\times (C/2)\times H\times W}$. To capture salient spatial regions at the patch level, *x*_*l*_ is unfolded into non-overlapping k×k patches, where *k* is typically set to 3 or 5. Although the unfolding operates in the local spatial domain, the filter coefficients applied to each patch are derived from the global descriptor, enabling each patch to be processed with awareness of the overall image structure.

A softmax function is then computed over the *k*^2^ positions within each patch to produce attention weights. Specifically, the softmax normalizes the learned filter generated from global context rather than raw pixel intensities, thereby implementing dynamic convolution with global information. These patch-wise attention weights are applied to *x*_*l*_ to yield an attention-weighted low-frequency map $x_{l_{a}}\in {\mathbb{R}}^{B\times (C/2)\times H\times W}$ that highlights coherent, large-scale structures [35,36].

To compute the high-frequency component, $x_{l_{a}}$ is first projected back to the original channel dimension *C* using a 1×1 convolution, producing ${\tilde{x}}_{l_{a}}\in {\mathbb{R}}^{B\times C\times H\times W}$. The high-frequency residue is then obtained as:


$$x_{h}=x-{\tilde{x}}_{l_{a}}.$$ (1)

Both $x_{l_{a}}$ and *x*_*h*_ are processed in parallel by small 1×1 convolutional blocks to produce per-stream attention vectors *a*_*l*_ and *a*_*h*_, respectively. These vectors are normalized and applied element-wise to their corresponding streams to emphasize the most relevant features in each frequency domain. The weighted low- and high-frequency streams are then summed and passed through a final 1×1 convolution to restore the original channel dimension, producing the fused output $x_{f}\in {\mathbb{R}}^{B\times C\times H\times W}$. This fusion integrates global and local cues into a single, spatially coherent representation while keeping the module’s complexity proportional to *HW*. Due to this dynamic convolution formulation, the module achieves a computational complexity of $O(N\times C\times k^{2}\times H\times W)$. In comparison, a multi-head self-attention mechanism with the same number of parameters incurs approximately four times more FLOPs.

### 3.3 ContextGate block

**Fig 3:**
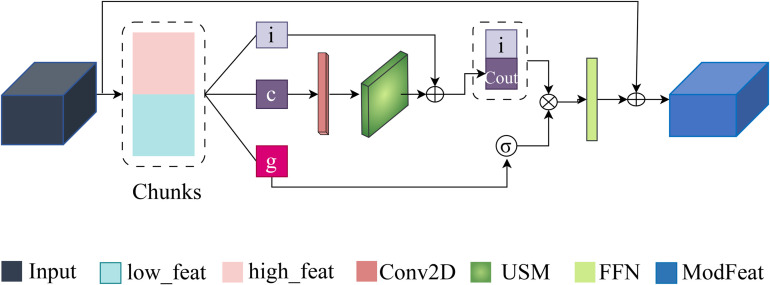
ContextGate block with three pathways: spatial (depthwise conv + SpectralFlow), gating (pointwise conv + GELU), and identity (pointwise conv).

The ContextGate block, demonstrated in Fig 3, provides a dynamic and task-aware modulation of features by learning spatial and channel-wise dependencies that indicate clinical relevance. Rather than treating all features equally, ContextGate adaptively scales and fuses components of the feature map.

For incoming features $x\in {\mathbb{R}}^{B\times C\times H\times W}$, the block first extracts localized spatial patterns (e.g., *i*, *c*, and *g*) via a depthwise convolution with a 3×3 kernel; this spatial processing captures per-channel texture and common micro-patterns. The spatial output (e.*g*., *c*) is then refined by the USM to ensure that both local detail and broader context are represented coherently; we denote the refined spatial output as *c*_out_ with dimension B×(C/3)×H×W. In parallel, a gating pathway applies a pointwise convolution to reduce the channel dimensionality to *C*/3 and then passes the result through a GELU nonlinearity to produce element-wise gating weights $g\in [0,1{]}^{B\times (C/3)\times H\times W}$. These weights indicate the relevance of spatial positions and channels for the target classification task. At the same time, an identity pathway preserves complementary information from the input via a pointwise convolution that outputs $i\in {\mathbb{R}}^{B\times (C/3)\times H\times W}$.

The block then concatenates the refined spatial representation *c*_out_ and the identity projection *i* along the channel axis to form a 2*C*/3-channel intermediate, which is multiplicatively modulated by the gating tensor *g* [37,38] as described in (2). After this gating operation, a final pointwise convolution projects the result back to the original *C* channels, yielding $x_{\text{out}}\in {\mathbb{R}}^{B\times C\times H\times W}$. To maintain stable gradients and preserve the backbone signal, the ContextGate output is added residually to the original input as in (3).


$$x_{\text{mod}}=g⊙\text{Concat}(c_{\text{out}},i)$$ (2)

$$x_{\text{final}}=x_{\text{out}}+x$$ (3)

This residual addition ensures that the block refines rather than overrides information, improving robustness and convergence during training. In practice, ContextGate focuses network capacity on clinically informative features and reduces sensitivity to spurious noise and imaging artifacts.

## 4 Experimental results & analysis

In this section, we present the experimental results of our proposed MedSpectralNet model and analyze them in comparison to state-of-the-art benchmark models.

### 4.1 Datasets and preprocessing

Six datasets from MedMNIST [13] have been used to investigate the performance of our proposed model; these are: BloodMNIST, BreastMNIST, DermaMNIST, PneumoniaMNIST, OrganCMNIST and OrganSMNIST. Among them, BloodMNIST consists of individual normal blood cells captured from healthy individuals, organized into 8 classes. It has 17,092 microscope images used for multi-class classification. Similarly, BreastMNIST is based on breast ultrasound images classified mainly into normal, benign, and malignant classes. PneumoniaMNIST focuses on binary classification of chest X-ray images to detect pneumonia. On the other hand, OrganCMNIST is a dataset from the MedMNIST v2 collection, specifically designed for multi-class classification of colored abdominal organ images. Lastly, the OrganSMNIST dataset is derived from abdominal CT scans and focuses on multi-class organ classification.

To ensure consistent model convergence, all input images were normalized using the standard ImageNet statistics. Specifically, pixel intensity values were normalized channel-wise using mean values of [0.485, 0.456, 0.406] and standard deviation values of [0.229, 0.224, 0.225] for the RGB channels, respectively. This standardization scheme accounts for the different intensity distributions inherent to various medical imaging modalities in the MedMNIST benchmark, effectively mapping diverse intensity ranges to a common normalized space that facilitates stable gradient flow during training.

Standard geometric augmentations were applied after processing, including random horizontal flipping, affine transformations, translation up to 10%, and perspective distortion. Color-based augmentations included ColorJitter to vary brightness, contrast, saturation, and hue, along with GaussianBlur for minor smoothing. To simulate occlusion, we applied RandomErasing with a 50% probability.

In addition to improving model generalization and robustness, we applied CutMix augmentation [39] on the datasets during training. It was employed with a probability of 0.5 during training, where two images and their labels are mixed by replacing a random patch of one image with a patch from another, effectively regularizing the model and encouraging it to attend to multiple regions.

### 4.2 Evaluation Metrics

To assess the performance of the proposed model, we employed standard evaluation metrics derived from the confusion matrix: true positives (*T*_*P*_), false negatives (*F*_*N*_), false positives (*F*_*P*_), and true negatives (*T*_*N*_). These quantities characterize the outcomes of binary classification tasks and form the basis of the following three measures.

**Receiver Operating Characteristic (ROC) and Area Under the ROC Curve (AUC):** The ROC curve illustrates the diagnostic ability of a classifier by plotting the True Positive Rate (TPR) against the False Positive Rate (FPR) across different thresholds, where


$$\text{TPR}=\frac{T_{P}}{T_{P}+F_{N}}$$ (4)

$$\text{FPR}=\frac{F_{P}}{F_{P}+T_{N}}$$ (5)

Consequently, the AUC quantifies overall discriminative ability, with higher values indicating stronger performance. In medical imaging applications, AUC represents the probability that the model will rank a randomly chosen positive case like malignant lesion higher than a randomly chosen negative case (e.g., benign or normal tissue). An AUC of 0.5 indicates random performance, while AUC = 1.0 represents perfect discrimination.

**Precision-Recall Curve (PRC):** Particularly useful for imbalanced datasets, the PRC evaluates a model in terms of Precision and Recall, defined as


$$\text{Precision}=\frac{T_{P}}{T_{P}+F_{P}}$$ (6)

$$\text{Recall}=\frac{T_{P}}{T_{P}+F_{N}}.$$ (7)

The area under the PR-AUC curve summarizes the trade-off between precision and recall.

The precision-recall curve is particularly valuable in medical imaging where class imbalance is common. Precision determines the probability that a model-flagged case is truly positive, directly affecting follow-up testing costs and patient anxiety. High precision reduces unnecessary procedures. Conversely, recall or sensitivity determines the proportion of diseased patients correctly identified, which is critical for minimizing missed diagnoses. For imbalanced datasets where disease prevalence is low (<5%), PR-AUC provides more informative assessment than ROC-AUC by emphasizing minority class performance.

**Accuracy:** Accuracy measures the proportion of correct predictions over all cases and is given by


$$\text{Accuracy}=\frac{T_{P}+T_{N}}{T_{P}+T_{N}+F_{P}+F_{N}}.$$ (8)

These metrics together provide a comprehensive evaluation of classification performance, capturing overall correctness as well as the discriminative power and robustness under class imbalance.

**FLOPs:** FLOPs quantify the computational complexity of a neural network by counting the total number of arithmetic operations required for a single forward pass through the network. FLOPs serve as a hardware-agnostic metric for evaluating model efficiency, as they measure theoretical computational cost independent of specific hardware implementations or software optimizations. The total network FLOPs aggregate contributions from all layers. Lower FLOPs indicate reduced computational burden, enabling faster inference and lower energy consumption are critical factors for deployment in resource-constrained clinical environments.

### 4.3 Results

In this section, we present a comprehensive evaluation of the proposed model on selected MedMNIST datasets and compare its performance against several established lightweight architectures, each of which has demonstrated strong results in downstream medical image classification tasks. Among the six selected datasets, OrganCMNIST and OrganSMNIST contain the largest number of samples. Given their substantial data size, we begin our investigation by evaluating MedSpectralNet’s performance on these two datasets as representative examples. FLOPs were measured using the thop profiling library with standardized input resolution of 224×224 pixels.

#### 4.3.1 OrganCMNIST dataset.

**Fig 4:**
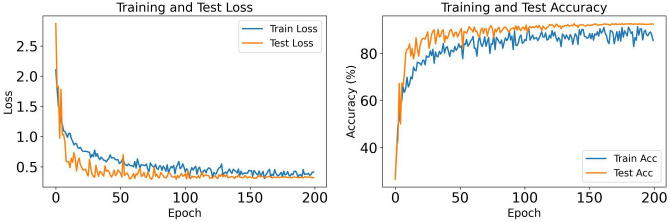
Training and test loss (left) and accuracy (right) curves for MedSpectralNet on OrganCMNIST dataset over 200 epochs.

Fig 4 presents the training dynamics for OrganCMNIST across 200 epochs. Both training and test losses decrease rapidly within the first 50 epochs and stabilize around epoch 100, with training loss converging to approximately 0.5 and test loss to 0.35. The consistent gap between curves indicates effective regularization without overfitting. The accuracy curves show steep improvement in the initial 50 epochs, reaching over 80% for both training and test sets. By epoch 100, performance plateaus around 93% test accuracy and approximately 90% training accuracy. The test accuracy exceeding training accuracy suggests strong generalization, confirming that the SpectralFlow modules effectively extract discriminative features for organ classification.

**Fig 5:**
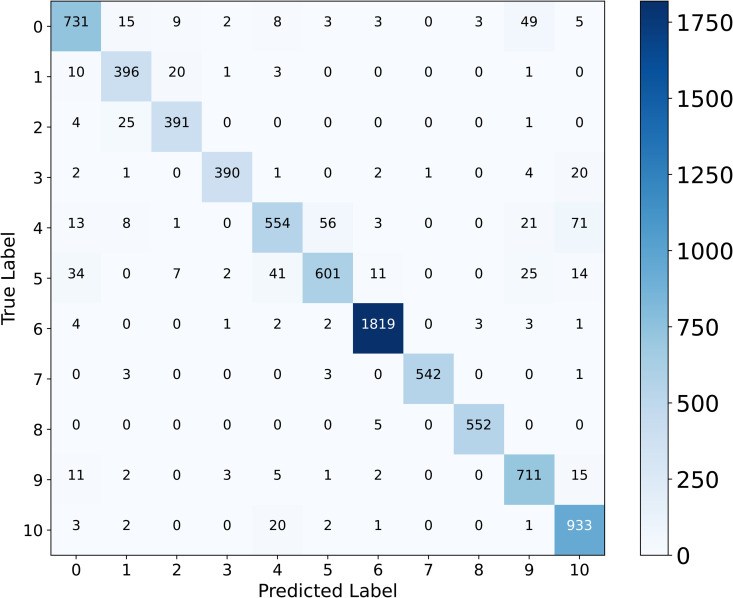
Confusion matrix for MedSpectralNet on OrganCMNIST dataset showing class-wise prediction distribution across 11 organ categories.

Fig 5 shows the confusion matrix, revealing strong performance across most organ classes. Class 6 achieves near-perfect classification with 1,819 correct predictions, demonstrating the model’s ability to capture highly distinctive organ features. Classes 0, 1, 2, 3, 7, 8, 9, and 10 also show solid performance with 731, 396, 391, 390, 542, 552, 711, and 933 correct predictions, respectively. However, several systematic misclassification patterns emerge among specific class pairs. Class 4 exhibits the most distributed confusion, with 71 samples misclassified as Class 10, 56 as Class 5, and 21 as Class 9. Class 5 shows reciprocal confusion with Class 4 (41 misclassifications) and moderate errors toward Class 0 (34 instances), indicating shared visual characteristics between these categories. Class 0 displays notable confusion with Class 9 (49 misclassifications), suggesting an overlapping appearance between these organ regions.

These error patterns reflect clinical challenges inherent to abdominal organ classification. The mutual confusion among Classes 4, 5, 9, and 10 corresponds to organs with similar tissue density or anatomical proximity, where overlapping morphological features create latent classification ambiguity. Class 0’s misclassification toward Class 9 similarly reflects anatomical adjacency in the imaging plane. Conversely, Class 6’s high accuracy suggests it represents an organ with distinguishable density and boundary characteristics that the model captures. The concentration of errors within anatomically related class pairs, rather than random distribution across all classes, demonstrates that the model learns meaningful organ relationships.

**Fig 6:**
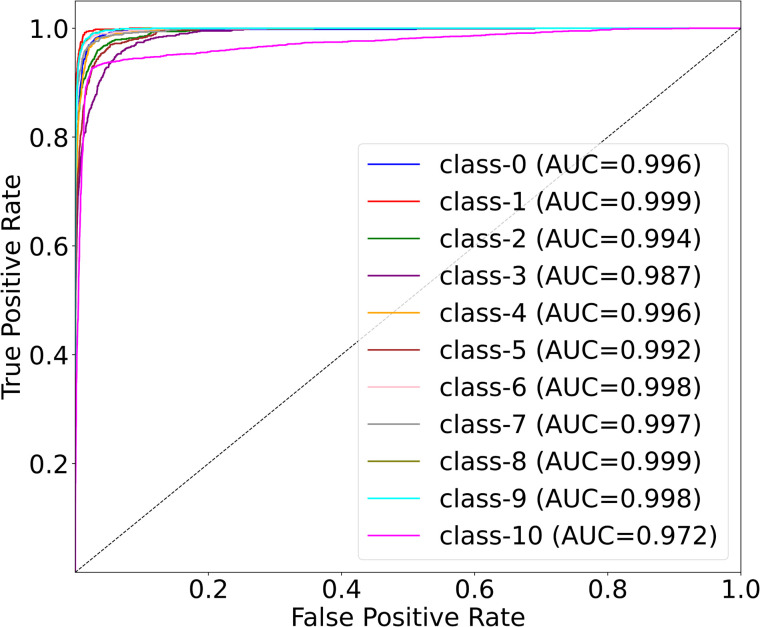
Multi-class ROC results of MedSpectralNet on the OrganCMNIST dataset.

Fig 6 presents the multi-class ROC curves for the OrganCMNIST dataset, illustrating the discriminative performance of MedSpectralNet across all 11 organ classes (Class 0 to Class 10). All curves are positioned near the top-left corner, significantly deviating from the random classifier baseline (black dashed line), which indicates strong classification capability. The corresponding AUC values range from 0.972 to 0.999. These results confirm that the proposed model maintains a consistently high TPR while minimizing FPR predictions across diverse organ categories. The small variation observed between classes (e.g., Class 3 and Class 10) suggests that while performance is nearly uniform, slight differences may arise from inter-class similarities or imbalances in the dataset. Nonetheless, the near-perfect AUC values across all eleven classes demonstrate that MedSpectralNet is highly effective at distinguishing between complex anatomical structures, even in challenging multi-class settings.

From a clinical perspective, this level of discriminative performance implies reliable diagnostic support in tasks involving multiple organ types, where misclassification could otherwise lead to ambiguous or misleading outcomes. The robustness of MedSpectralNet across all classes also highlights its potential for deployment in real-world applications, where consistency across heterogeneous data is as critical as overall accuracy.

**Fig 7:**
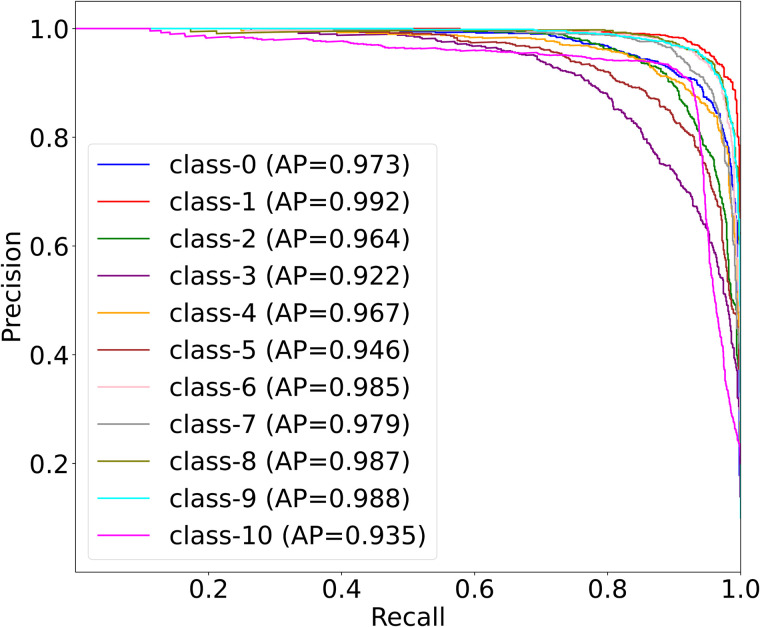
Multi-class PRC results of MedSpectralNet on the OrganCMNIST dataset.

Fig 7 presents the multi-class PRC results for applying MedSpectralNet on the OrganCMNIST dataset. Overall, the curves demonstrate that MedSpectralNet maintains high precision across a wide range of recall values, confirming its ability to correctly identify positive cases while minimizing false positives, with AUC values ranging from 0.922 to 0.992. The slightly lower values observed for Class 3 (0.922) and Class 10 (0.935) suggests that these categories present greater challenges. Nonetheless, even in these more difficult cases, performance remains well above acceptable thresholds, indicating strong robustness of the model. For the remaining classes, the near-perfect values highlight the model’s ability to consistently balance sensitivity and specificity.

The PRC analysis complements the ROC results by providing a more informative view under imbalanced conditions, where high accuracy alone may not reflect true discriminative ability. From a clinical standpoint, these findings suggest that MedSpectralNet can effectively prioritize the detection of organ-specific features while maintaining low rates of false alarms, which is crucial for building trust in diagnostic decision-support systems. Together with the ROC analysis, PRC confirms that the proposed architecture delivers both high overall performance and reliable class-wise stability across diverse organ categories. Overall, the proposed MedSpectralNet model achieved remarkable performance on the OrganCMNIST dataset, with an average accuracy of 93.7%, indicating its ability to effectively distinguish between different organ classes with strong predictive capability.

#### 4.3.2 OrganSMNIST dataset.

**Fig 8:**
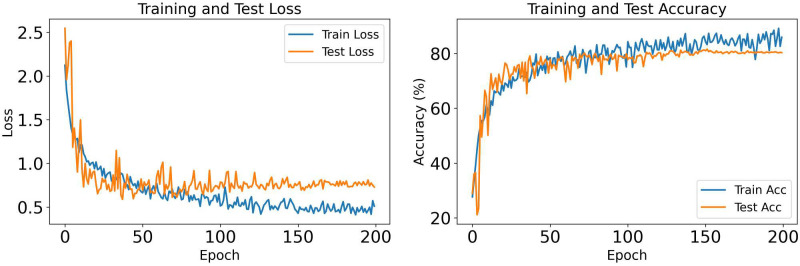
Training and test loss (left) and accuracy (right) curves for MedSpectralNet on OrganSMNIST dataset over 200 epochs.

Fig 8 presents the training dynamics for OrganSMNIST across 200 epochs. Both training and test losses converge rapidly in the first 50 epochs and stabilize around epoch 100, with training loss settling near 0.5 and test loss plateauing higher at approximately 0.8. The persistent gap between the two loss curves indicates mild overfitting, with the model generalizing reasonably but not perfectly to unseen data. Similarly, accuracy curves show steep improvement to 75–80% by epoch 50, with training accuracy continuing a gradual upward trend to approximately 85–87% by epoch 200, while test accuracy plateaus around 80–82%. The modest divergence between training and test metrics suggests the model achieves solid generalization performance, though some degree of overfitting is present in the later epochs.

**Fig 9:**
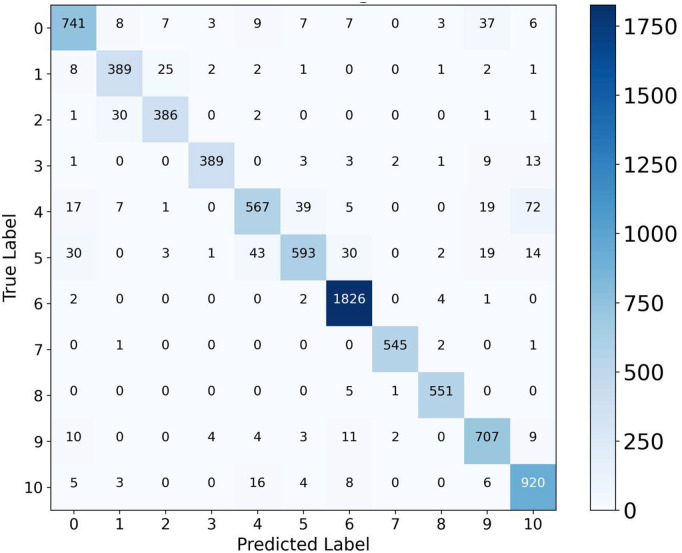
Confusion matrix for MedSpectralNet on OrganSMNIST dataset showing class-wise prediction distribution across 11 organ categories.

Fig 9 shows the confusion matrix, revealing systematic error patterns. Class 6 achieves near-perfect performance where 1,826 samples give correct predictions. Classes 0, 1, 2, 3, 7, and 8 demonstrate strong accuracy with a range of 389–741 correct predictions. However, Class 4 exhibits the broadest confusion, with 72 misclassifications as Class 10, 39 as Class 5, and 19 as Class 9. This indicates overlapping visual features due to similar intensity distributions in abdominal CT scans. Class 10 shows reciprocal confusion with Class 4 (72 instances) and moderate confusion with Classes 5 and 9, suggesting it occupies an intermediate position in feature space.

These error patterns reflect clinically relevant challenges. The mutual confusion between Classes 4, 5, 9, and 10 indicates anatomically adjacent organs with similar radiological appearances. This implies the diagnostic challenges radiologists face when organs exhibit overlapping Hounsfield unit ranges. Conversely, Class 6’s high performance suggests distinctive spectral characteristics that the dual-stream architecture effectively captures.

**Fig 10:**
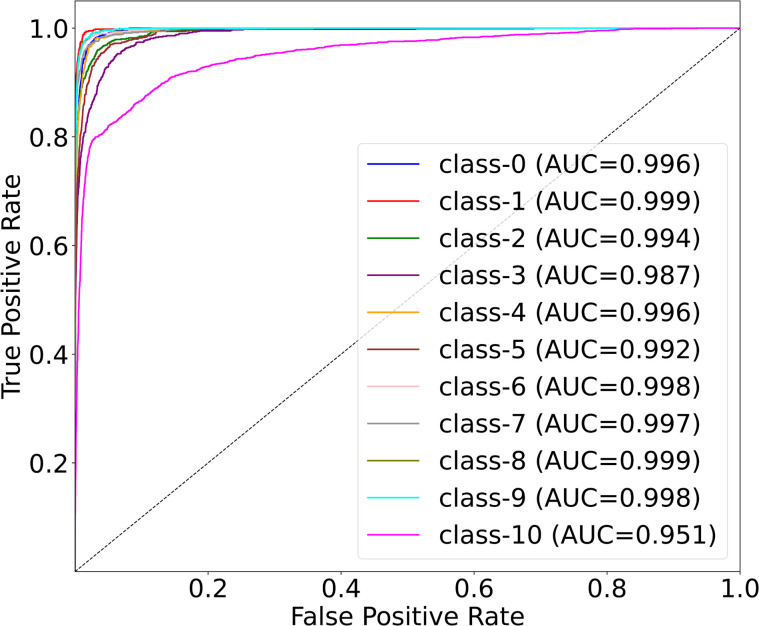
Multi-class ROC results of MedSpectralNet on the OrganSMNIST dataset.

Fig 10 shows the multi-class ROC curves for the OrganSMNIST dataset, summarizing MedSpectralNet’s discriminative ability across the 11 organ classes (Class 0 to Class 10). Again, we notice that all curves are positioned near the top-left corner, significantly deviating from the random classifier baseline, indicating robust classification capability similar to that of OrganCMNIST. The corresponding AUC values span a narrow, high range, demonstrating that MedSpectralNet reliably ranks positive examples above negatives for nearly every class across all decision thresholds. However, the variation observed across classes (e.g., Class 10 with an AUC of 0.951) suggests that, while performance is generally excellent, some classes may pose greater challenges. Nevertheless, the consistently high AUC values across all classes demonstrate that MedSpectralNet effectively distinguishes between complex anatomical structures in this challenging multi-class scenario on OrganSMNIST.

**Fig 11:**
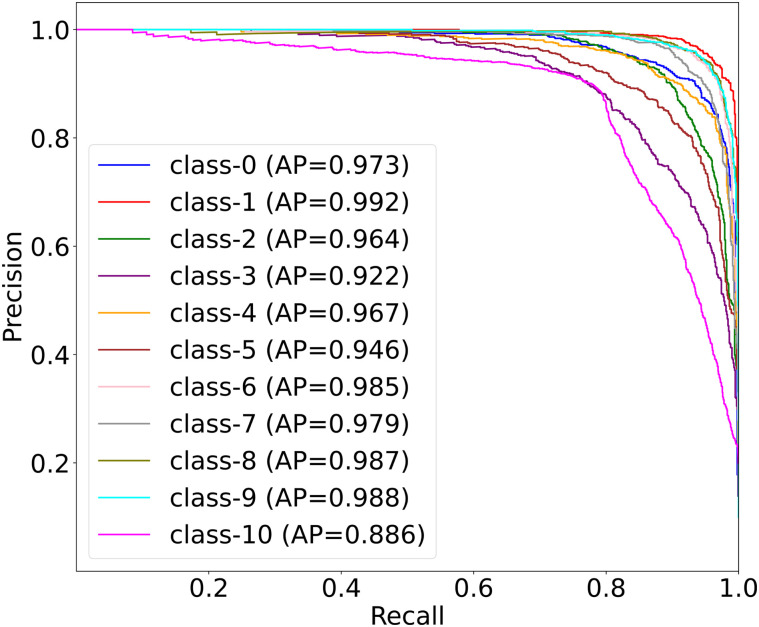
Multi-class PRC results of MedSpectralNet on the OrganSMNIST dataset.

On the other hand, Fig 11 reveals more nuanced performance characteristics through PRC analysis. The wide range of average precision values (0.886 to 0.992) suggests that MedSpectralNet exhibits class-dependent decision boundaries, where some organ types benefit from more robust feature extraction pipelines than others. This variability indicates that the model’s spectral feature learning mechanism may be more attuned to certain anatomical patterns, potentially reflecting the frequency domain characteristics inherent to different tissue types or imaging modalities. The consistent performance across most classes except for Class 10 suggests that the spectral network architecture has successfully learned generalizable organ-discriminative features, but encounters specific challenges with certain anatomical structures.

These results support several clinical applications. The high AUC values enable effective triage capabilities, allowing healthcare professionals to prioritize urgent cases while confidently ruling out pathologies in lower-risk patients. The balanced sensitivity across organ systems reduces systematic bias risks. For resource-limited settings, the model’s reliable automated classification could extend diagnostic capabilities to underserved areas, improving healthcare equity. Additionally, the consistent performance across most categories positions the system as a quality assurance tool, standardizing organ identification and reducing inter-observer variability across healthcare institutions.

### 4.4 Ablation study and grad-CAM analysis of BreastMNIST

To validate the effectiveness of our dual SpectralFlow architecture and provide interpretability insights, we conducted ablation study and Gradient-weighted Class Activation Mapping (Grad-CAM) analysis on both spectralflow modules and ContextGate module using the BreastMNIST dataset. To address the critical need for explainability in medical AI systems and validate the effectiveness of our architectural components, we conducted comprehensive Grad-CAM analysis on two key modules within our MedSpectralNet architecture: the ContextGate Block and SpectralFlow Module. These modules represent the core of our dual-stream feature processing pipeline, where parallel pathways converge to produce refined, diagnostically-relevant representations.

#### 4.4.1 ContextGate block: Adaptive multi-scale feature modulation.

**Fig 12:**
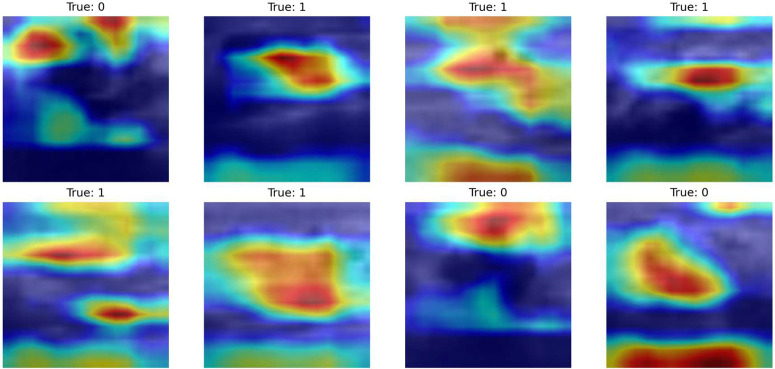
Grad-CAM visualization for ContextGate Block on BreastMNIST. Class 0 (normal) shows distributed attention suggesting holistic assessment, while Class 1 (abnormal) focuses on suspicious regions, reflecting adaptive gating behavior.

Fig 12 reveals the activation patterns from the ContextGate block’s depthwise convolution pathwaye. This module operates in parallel with the SpectralFlow pathway, implementing a three-pathway design: spatial processing (depthwise conv + SpectralFlow), gating mechanism (pointwise conv + GELU), and identity preservation (pointwise conv). The Grad-CAM visualizations demonstrate intermediate-level feature localization characterized by moderate activation clustering and emerging spatial selectivity.

The activation maps reveal structured patterns with identifiable regions of interest that bridge the gap between global contextual awareness and precise diagnostic localization. Unlike uniform global pooling or purely local convolutions, the ContextGate exhibits spatially-selective activation that adapts to image content. The heterogeneous activation patterns across samples demonstrate that the gating mechanism successfully adapts to varying tissue characteristics and pathological presentations. For normal tissue samples (Class 0), the activations tend to be more distributed across multiple regions, suggesting that normal classification relies on holistic structural assessment indicates overall tissue organization, echo patterns, and architectural integrity than focusing on isolated features. Conversely, for abnormal tissue samples (Class 1, encompassing both benign and malignant pathologies), the activation maps show emerging concentration toward specific suspicious regions, indicating that the ContextGate begins to prioritize areas with potential pathological significance.

A critical observation is that activation intensity and spatial distribution vary significantly between samples within the same class. This sample-specific modulation demonstrates that the ContextGate learns nuanced, adaptive filtering rather than applying fixed spatial templates. For instance, examining the Class 1 samples in Fig 12, we observe different activation patterns: some concentrate on central regions (row 1, columns 2–3), others emphasize peripheral structures (row 2, column 2), and some exhibit multi-focal activation (row 2, column 3).

Within our overall architecture, the ContextGate block processes the same input features as the first SpectralFlow module but through a fundamentally different pathway. While SpectralFlow performs frequency-domain decomposition into high and low components, ContextGate implements spatial-domain gating with learned attention. The outputs of these parallel pathways are subsequently fused through residual connections, enabling the network to leverage complementary information: spectral decomposition captures multi-frequency patterns essential for texture analysis, while contextual gating emphasizes spatially-coherent structures important for anatomical understanding.

#### 4.4.2 SpectralFlow module.

**Fig 13:**
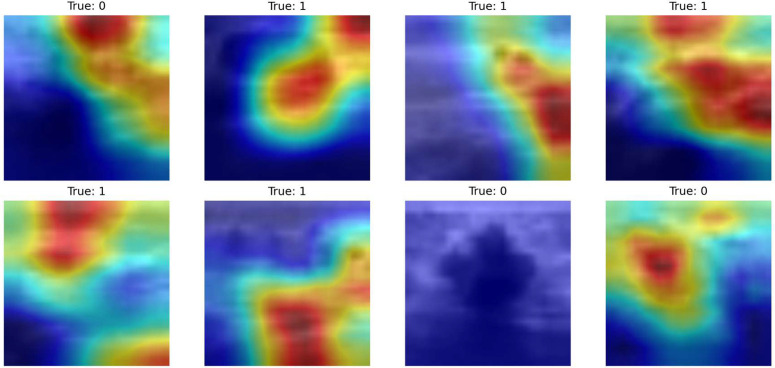
Grad-CAM for SpectralFlow Module on BreastMNIST. Visualizations show precise, localized activations on diagnostically relevant regions in Class 1, while Class 0 displays more distributed patterns.

Fig 13 reveals significantly more focused and localized activation patterns from the second SpectralFlow module, which operates after the ContextGate block, depthwise convolution, and residual fusion stages. This module represents the final feature refinement stage before global average pooling and classification. The heatmaps display distinct high-activation regions (red and yellow areas) sharply concentrated on specific spatial locations within the ultrasound images, demonstrating successful diagnostic feature localization.

The sharp gradients between high-activation zones (red/yellow) and low-activation zones (blue/purple) indicate that SpectralFlow Module has learned precise spatial localization capabilities essential for medical image classification. Unlike the more diffuse patterns observed in the ContextGate, SpectralFlow exhibits focused attention peaks that correspond to diagnostically relevant anatomical structures and potential pathological features.

For abnormal tissue samples (Class 1), the concentrated high-intensity regions align with the types of features that radiologists examine during ultrasound interpretation: mass boundaries and margins (regularity vs. irregularity), internal echo patterns (homogeneous vs. heterogeneous), posterior acoustic phenomena (enhancement vs. shadowing), and architectural distortions.

Examining class-specific activation patterns reveals the model’s decision-making strategy. For most Class 0 samples (normal tissue), the second SpectralFlow module exhibits distributed activation patterns across multiple regions rather than sharp focal peaks. This distributed attention suggests that normal tissue classification relies on holistic structural assessment, that evaluates overall tissue organization, echo uniformity, and absence of suspicious focal findings, rather than identifying specific isolated features. This aligns with clinical practice, where normality is often determined by the absence of abnormalities and preservation of expected anatomical patterns.

Conversely, for Class 1 samples (abnormal tissue), the activation maps show concentrated high-intensity regions that indicate focused examination of specific suspicious areas. These focal activations correspond to pathological indicators such as mass boundaries, echogenic irregularities, or architectural distortions. The activation intensity varies significantly across different abnormal samples, reflecting the heterogeneous morphological presentations of benign and malignant lesions. Some samples exhibit single strong activation peaks (row 1, column 2), while others show multiple activation foci (row 2, column 3), appropriately adapting to multi-focal or diffuse pathological patterns.

This class-dependent activation behavior which is distributed for normal, focused for abnormal class, mirrors expert radiological assessment strategies and validates that the model has learned clinically appropriate decision boundaries rather than relying on spurious correlations or dataset-specific biases.

A critical observation emerges in sample 6 (row 2, column 2) labeled as Class 0 (normal tissue), which exhibits anomalously weak activation across the entire spatial extent, appearing as predominantly dark blue coloring throughout. This activation failure represents a notable exception to the otherwise consistent activation patterns and warrants careful interpretation. From a clinical deployment perspective, such activation failures should not be viewed solely as model limitations but rather as valuable uncertainty indicators that can enhance safety and reliability.

The Grad-CAM results empirically demonstrate that this efficient convolutional approximation successfully captures the multi-scale, spatially-adaptive attention essential for medical image understanding. The progression from emerging localization (ContextGate) to precise focusing (SpectralFlow) shows that our architecture learns hierarchical feature representations comparable to transformer-based approaches while requiring substantially fewer parameters (8.5M vs. 14.5M for MedMamba-T) and maintaining better parallelization on modern GPU hardware.

### 4.5 Ablation study on BreastMNIST

We conducted a comprehensive ablation study on the BreastMNIST dataset based on the optimization algorithm and the data augmentation strategy. All ablation experiments were conducted under identical environmental settings to ensure fair comparison. Under normal augmentation setting, we applied only standard resizing, random horizontal flipping, and normalization. We evaluated three different optimizers: Stochastic Gradient Descent (SGD), RMSprop, and Adam. Additionally, we analyzed the performance gain attributed specifically to the CutMix augmentation strategy. The quantitative results are presented in Table 2.

**Table 2:** Ablation study of different optimizers and augmentation strategies on the BreastMNIST dataset. The best performance is highlighted in bold.

Experiment	Optimizer	Augmentation	Accuracy (%)	AUC
Baseline	Adam	Normal	84.2±0.8	0.885
–	SGD	CutMix	83.1±1.2	0.864
–	RMSprop	CutMix	84.9±0.9	0.891
**Proposed**	**Adam**	**CutMix**	86.5±0.5	**0.912**

### 4.6 Evaluation result

Table 3 presents comprehensive evaluation results of our proposed model on the selective MedMNIST dataset to some reference models that demonstrated excellent performance in their respective architectures in downstream tasks. As shown, MedSpectralNet achieves competitive results with state-of-the-art medical image classification models while maintaining notable computational efficiency. In summary, MedSpectralNet offers 93.7% accuracy on OrganCMNIST, 98.0% accuracy on BloodMNIST, and strong performance on BreastMNIST with 86.5%, DermaMNIST with 78.4%, OrganSMNIST with 83.1%, and PneumoniaMNIST with 94.2% accuracy. These results position MedSpectralNet favorably against MedMamba-T and other established benchmarks.

**Table 3:** Accuracy comparison of MedSpectralNet with benchmark models across selected datasets.

Model	PneumoniaMNIST	BreastMNIST	BloodMNIST	DermaMNIST	OrganCMNIST	OrganSMNIST
ResNet18 (28) [31]	0.854	0.863	0.958	0.735	0.900	0.782
ResNet18 (224) [31]	0.864	0.833	0.963	0.754	0.920	0.778
ResNet50 (28) [31]	0.854	0.812	0.956	0.735	0.905	0.770
ResNet50 (224) [31]	0.884	0.842	0.950	0.731	0.911	0.785
Auto-sklearn [40]	0.855	0.803	0.878	0.719	0.829	0.672
AutoKeras [41]	0.878	0.831	0.961	0.749	0.879	0.813
Google AutoML [42]	0.946	0.861	0.966	0.768	0.877	0.749
MedViT-T [24]	0.949	0.896	0.950	0.768	0.901	0.789
MedViT-S [24]	0.961	0.897	0.951	0.780	0.916	0.805
MedViT-L [24]	0.921	0.883	0.954	0.773	0.922	0.806
MedMamba-S [6]	0.936	0.853	0.984	0.758	0.944	0.833
MedMamba-B [6]	0.925	0.891	0.983	0.757	0.943	0.834
MedMamba-T [6]	0.899	0.853	0.978	0.779	0.927	0.819
**MedSpectralNet**	**0.942**	**0.865**	**0.980**	**0.784**	**0.937**	**0.831**

MedSpectralNet consistently outperforms the MedMamba-T baseline across all six datasets, with particularly notable improvements on PneumoniaMNIST, achieving a 4.3% improvement, OrganCMNIST with 1.0% gain, and OrganSMNIST with 1.2% enhancement. While our model underperforms compared to MedMamba-S and MedMamba-B on some datasets, they require significantly more computational resources with substantially larger parameter counts. The consistency of improvements across all datasets indicates that MedSpectralNet’s architectural advantages are not dataset-specific but represent fundamental improvements in feature extraction and representation learning through spectral decomposition.

When compared to medical-specific ViTs in the MedViT family, MedSpectralNet demonstrates competitive performance while maintaining substantial parameter efficiency advantages. On PneumoniaMNIST, our model achieves 94.2% accuracy, closely matching MedViT-S at 96.1% and MedViT-T at 94.9% but with significantly fewer parameters. The performance gap narrows considerably on other datasets, with MedSpectralNet achieving comparable or superior results on BreastMNIST at 86.5% versus MedViT variants achieving 89.6% to 89.7%, and BloodMNIST at 98.0% versus MedViT variants achieving 95.0% to 95.4%.

In the case of traditional CNN architecture, MedSpectralNet demonstrates comparatively superior performance across all datasets. On PneumoniaMNIST, it achieves 94.2% accuracy compared to ResNet18’s performance of 85.4% on 28×28 resolution and 86.4% on 224×224 resolution, as well as ResNet50’s results of 85.4% on 28×28 and 88.4% on 224×224. This represents substantial improvements of 8.8%, 7.8%, 8.8%, and 5.8%, respectively.

When compared to automated machine learning approaches, MedSpectralNet shows substantial improvements across all evaluated datasets. The model outperforms Auto-sklearn by significant margins, achieving 8.7% improvement on PneumoniaMNIST, 6.2% on BreastMNIST, 10.2% on BloodMNIST, 6.5% on DermaMNIST, 10.8% on OrganCMNIST, and 15.9% on OrganSMNIST. Against AutoKeras and Google AutoML, MedSpectralNet maintains competitive performance while offering greater architectural transparency and interpretability for medical applications.

To substantiate claims of computational efficiency and real-time suitability for clinical deployment, we conducted a comprehensive analysis of floating-point operations (FLOPs) and inference performance. This analysis addresses the critical need for lightweight architectures in medical imaging, where computational resources are often constrained yet diagnostic accuracy cannot be compromised.

From a clinical perspective, the consistent performance across 2D-MedMNIST benchmarks indicates high diagnostic robustness. A high AUC in OrganCMNIST (93.7%) suggests that MedSpectralNet can reliably assist radiologists in multi-organ segmentation and classification, reducing the manual screening burden and potentially lowering the diagnostic error rate in high-volume clinical settings.

### 4.7 Computational efficiency

#### 4.7.1 FLOPs comparison.

Table 4 presents the computational cost comparison between MedSpectralNet and state-of-the-art architectures evaluated on medical imaging tasks.

**Table 4:** Computational efficiency comparison on medical image classification. FLOPs measured in giga floating-point operations (G).

Model	Params (M)	FLOPs (G)
ResNet-18 [31]	11.7	1.81
ResNet-50 [31]	25.6	4.09
Swin-T [43]	27.5	4.50
ConvNeXt-T [44]	27.8	4.47
InceptionMamba [45]	7.0	0.80
**MedSpectralNet (Ours)**	**8.5**	**2.00**

MedSpectralNet achieves 2.00 GFLOPs, positioning it favorably within the spectrum of efficient medical imaging architectures. Against general-purpose vision models, the efficiency advantage is more pronounced: MedSpectralNet requires 55.6% fewer FLOPs than ConvNeXt-T (4.47 GFLOPs) and 55.1% fewer than Swin-T (4.50 GFLOPs).

#### 4.7.2 Parameter count.

**Fig 14:**
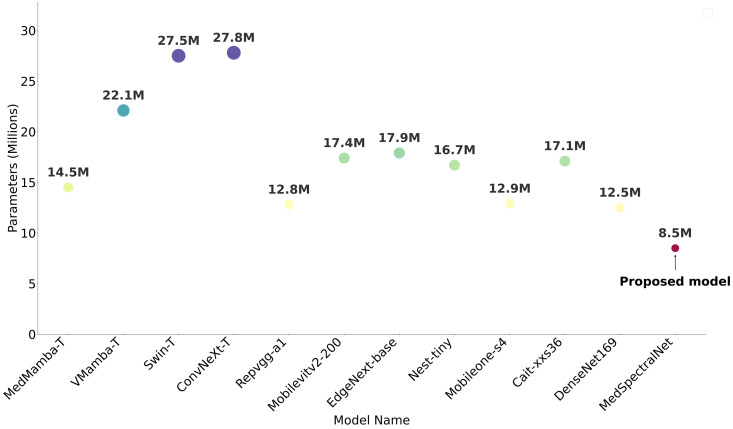
Parameter size required by MedSpectralNet in comparison to benchmark models.

The most compelling aspect of our findings lies in MedSpectralNet’s parameter efficiency. Fig 14 compares MedSpectralNet with several lightweight models, including MedMamba, V-Mamba, Swin Transformer, MobileViT, and Nest-Tiny [6,43,44,46–48]. With only 8.5M parameters, our model achieves performance comparable to MedMamba-T (14.5M parameters) and substantially outperforms other lightweight alternatives like VMamba-T (22.1M) and Swin-T (27.5M). This 40–60% reduction in model size directly translates to practical deployment advantages in resource-constrained clinical environments. Unlike MedMamba’s sequential state-space operations that limit GPU parallelization, our convolutional architecture fully leverages modern parallel computing hardware, resulting in faster inference times critical for real-time diagnostic applications.

This parameter efficiency translates to several practical advantages. The reduced GPU memory requirements enable deployment on edge devices, while faster convergence leads to reduced training time. Lower computational overhead facilitates real-time applications, and the reduced power requirements support sustainable deployment in resource-constrained environments.

The spectral decomposition approach used in MedSpectralNet offers several advantages over traditional spatial-domain methods. These efficiency gains are reflected not only in the reduced parameter count, as discussed earlier, but also in lower computational complexity. The linear *O*(*HW*) computational complexity of our SpectralFlow module represents a significant advancement over the ViTs quadratic complexity. This is particularly relevant for high-resolution medical imaging, where computational constraints often force trade-offs between image quality and processing speed.

Unlike MedMamba’s sequential state-space operations that limit GPU parallelization, our convolutional architecture fully leverages modern parallel computing hardware [4,5,39]. This architectural choice results in faster inference times that are critical for real-time diagnostic applications. The design provides better scalability across different hardware configurations and enables more efficient batch processing capabilities, ultimately leading to enhanced throughput for clinical workflows.

Overall, the combination of high performance and low parameter count makes the proposed MedSpectralNet particularly suitable for deployment in resource-constrained clinical environments. MedSpectralNet maintains a computational budget of 2.0 GFLOPs. This is significantly lower than the 4.0 + GFLOPs typically seen in ResNet-50. This efficiency facilitates the deployment of AI-driven diagnostics on low-power ARM-based mobile devices, bridging the gap between high-end laboratory research and bedside patient care. As a result, model can be effectively integrated into mobile diagnostic devices, edge computing applications in rural healthcare settings, real-time monitoring systems, and point-of-care diagnostic tools. This versatility addresses critical healthcare delivery challenges in underserved regions where computational resources are limited.

### 4.8 Limitations

While MedSpectralNet demonstrates strong performance across multiple benchmark datasets and offers substantial computational advantages, it is important to acknowledge its limitations and boundary conditions. The MedMNIST benchmark datasets used in our evaluation exhibit moderate class imbalance. However, severe class imbalance common in rare disease detection where positive cases may constitute <1% of samples, was not extensively evaluated in this study. Furthermore, handling extremely noisy or corrupted medical images remains an Out-of-Distribution (OOD) challenge.

## 5 Conclusions

This study presents MedSpectralNet, a lightweight convolutional architecture that successfully addresses the computational challenges of medical image classification while maintaining competitive diagnostic accuracy. Extensive evaluation across six multimodal benchmark datasets shows that, with only 8.5M parameters, MedSpectralNet achieves comparable or superior performance to MedMamba-T’s 14.5M parameters. Consequently, MedSpectralNet achieves equivalent results while using 60% fewer parameters than large state-of-the-art models, demonstrating significantly higher efficiency while preserving comparable performance. One critical observation from our study is that parameter efficient convolutional models can not only exceed benchmark performances of transformer alternatives, but also achieve global modeling at linear complexity. Future work will focus on extending the framework to 3D volumetric imaging, enhancing interpretability for clinical decision support, and conducting comprehensive validation across diverse clinical datasets to establish real-world efficacy and safety profiles. Additionally, investigating the integration of multimodal data sources could further enhance the models’ diagnostic accuracy. Future study will explore the integration of self-supervised pre-training to improve robustness against such artifacts.
